# Diagnostic and Prognostic Value of Serum Leptin in Critically Ill Patients with Acute versus Acute-on-Chronic Liver Failure

**DOI:** 10.3390/biomedicines12061170

**Published:** 2024-05-24

**Authors:** Philipp Hohlstein, Can Salvarcioglu, Maike R. Pollmanns, Jule K. Adams, Samira Abu Jhaisha, Elena Kabak, Albrecht Eisert, Karim Hamesch, Ralf Weiskirchen, Alexander Koch, Theresa H. Wirtz

**Affiliations:** 1 Department for Gastroenterology, Metabolic Disorders and Intensive Care Medicine, RWTH-University Hospital Aachen, Pauwelsstraße 30, 52074 Aachen, Germany; phohlstein@ukaachen.de (P.H.); can.salvarcioglu@rwth-aachen.de (C.S.); mpollmanns@ukaachen.de (M.R.P.); jadams@ukaachen.de (J.K.A.); sabujhaisha@ukaachen.de (S.A.J.); ekabak@ukaachen.de (E.K.); khamesch@ukaachen.de (K.H.); thwirtz@ukaachen.de (T.H.W.); 2 Hospital Pharmacy, RWTH-University Hospital Aachen, Pauwelsstraße 30, 52074 Aachen, Germany; aeisert@ukaachen.de; 3 Institute of Clinical Pharmacology, RWTH-University Hospital Aachen, Pauwelsstraße 30, 52074 Aachen, Germany; 4 Institute of Molecular Pathobiochemistry, Experimental Gene Therapy and Clinical Chemistry (IFMPEGKC), RWTH-University Hospital Aachen, Pauwelsstraße 30, 52074 Aachen, Germany; rweiskirchen@ukaachen.de

**Keywords:** leptin, adipokine, intensive care unit (ICU), critical illness, acute liver failure (ALF), acute-on-chronic liver failure (ACLF), human, biomarker, diagnosis, liver transplantation

## Abstract

Differentiation between acute liver failure (ALF) and acute-on-chronic liver failure (ACLF) can be challenging in patients with de novo liver disease but is important to indicate the referral to a transplant center and urgency of organ allocation. Leptin, an adipocyte-derived cytokine that regulates energy storage and satiety, has multiple regulatory functions in the liver. We enrolled 160 critically ill patients with liver disease and 20 healthy individuals to measure serum leptin concentrations as a potential biomarker for diagnostic and prognostic purposes. Notably, patients with ALF had higher concentrations of serum leptin compared to patients with decompensated advanced chronic liver disease (dACLD) or ACLF (110 vs. 50 vs. 29 pg/mL, *p* < 0.001). Levels of serum leptin below 56 pg/mL excluded ALF in patients with acute hepatic disease, with a negative predictive value (NPV) of 98.8% in our cohort. Lastly, serum leptin did not show any dynamic changes within the first 48 h of ICU treatment, especially not in comparison with patients with ALF vs. ACLF or survivors vs. non-survivors. In conclusion, serum leptin may represent a helpful biomarker to exclude ALF in critically ill patients who present with acute liver dysfunction.

## 1. Introduction

Acute (ALF) and acute-on-chronic liver failure (ACLF) are two distinct syndromes characterized by acute hepatic dysfunction, often necessitating treatment in the intensive care unit (ICU) and/or liver transplantation (LT). ALF is characterized by acute abnormal liver blood tests in patients without pre-existing chronic liver disease. ALF is a rare diagnosis, defined as acute liver injury (ALI) with impaired liver function measured by an international normalized ratio (INR) exceeding 1.5 and the occurrence of hepatic encephalopathy (HE) in patients without a chronic liver disease. In contrast, ACLF describes a severe form of acutely decompensated chronic liver disease with organ failure and high short-term mortality and occurs in patients with underlying chronic liver disease with the functional failure of at least one of six major organ systems (i.e., liver, kidney, brain, coagulation, circulation, or respiration) and systematic inflammation. Early categorization of acute liver dysfunction into ALF or ACLF is crucial for planning further diagnostic and therapeutic measures, but can be challenging, especially in patients without prior presentation to health care providers. Currently, there are no specific biomarkers to differentiate ALF from ACLF or to predict prognosis in these syndromes, highlighting the need for further research in this area [1,2,3,4,5,6].

The presence of a gene involved in the regulation of body fat was first suggested in the early 1950s by the discovery of the obese (ob/ob) mutation in mice which suffered from hyperphagia resulting in massive early obesity [7,8]. Much later, in 1995, the leptin receptor (LEPR) was described [9]. Leptin was found to be expressed in adipose tissue and released by adipocytes into the circulation for signaling purposes. Hence, the adipokine is a reliable biomarker for body fat content due to its strong correlation with adipose tissue mass [10,11]. The mechanistic pathway underlying the relationship between leptin and body mass index (BMI) may involve the transcription factor GATA3 acting as an immunomodulator. GATA3 is expressed in omental adipocytes of obese patients in this context, leading to hepatic inflammation via leptin and interleukin 6 signaling [12]. Furthermore, circulating leptin is positively correlated with female gender, likely due to differences in body fat distribution [13]. Short-term negative energy balance results in a quick decrease in leptin gene expression, but an acute increase in caloric balance only gradually restores leptin gene expression proportionally to fat mass [10,14]. In obesity, individuals develop a diminished response to leptin, resulting in “leptin resistance” [15]. Regarding hormonal regulations, insulin and glucocorticoids increase leptin mRNA levels, while catecholamines decrease them [10]. Leptin is a 4-helix bundle cytokine, with two molecules binding to the extracellular domain of a leptin receptor homodimer, activating the JAK2-STAT3 (Janus kinase–signal transducer and activator of transcription 3) signaling cascade [10]. The LEPR is mainly expressed in the central nervous system in hypothalamic neurons (Agoutí-related protein (AGRP)/neuropeptide Y (NPY) and pro-opiomelanocortin (POMC) neurons), which signal to second-order neurons to inhibit food intake as a “satiety factor” [10,16,17,18,19]. Other physiological effects of leptin include the regulation of lipid and glucose metabolism, fertility, and the immune system [20,21,22,23].

Adipokines, including leptin, have recently become of interest in the field of liver function and disease, as many affected metabolic pathways are closely linked to the liver [24,25]. The physiological effects of leptin on the liver are mediated through direct mechanisms (LEPR on hepatocytes) or indirect mechanisms (e.g., through the central nervous system) [24,25,26,27]. Leptin upregulates insulin sensitivity, β-oxidation, immune response and inflammatory mediators, angiogenesis, fibrogenesis, and tissue remodeling in the liver [24]. Moreover, the involvement of leptin has been described in several hepatic diseases including hepatic steatosis such as metabolic dysfunction-associated steatotic liver disease (MASLD) and metabolic dysfunction-associated steatohepatitis (MASH) [28], as well as hepatocellular carcinoma (HCC), alcoholic liver injury, chronic viral hepatitis, and parasitic infections [24]. Leptin stimulates the production of GATA3 in hepatic stellate cells, which may ultimately lead to the activation of NF-κBp65 and Kupffer cells with a predominant M1 macrophage phenotype, thereby contributing to the development of MASLD and insulin resistance [12]. Leptin also appears to be necessary for the development of hepatic fibrosis in response to chronic liver injury [29,30]. In lipodystrophy, MASLD, and MASH, leptin has been successfully used in treatment [31]. Elevated levels of leptin in the plasma have been associated with inflammatory and fibrogenic processes [32,33], as well as potential oncogenic effects on the liver [34]. An interesting finding is impaired liver regeneration after partial hepatectomy in leptin-deficient mice [35], suggesting that leptin signaling plays a significant role in the overall hepatic regeneration process [24]. Similarly, administration of intraperitoneal leptin before partial hepatectomy in rats improves final liver weight and histopathological damage [36]. In human liver donors, higher serum levels of leptin in the early phase after hepatectomy have been associated with accelerated liver regeneration [37], further highlighting the role of leptin in regeneration after liver damage. In critical illness, circulating leptin levels have been found to be increased in sepsis, but do not provide any additional diagnostic or prognostic value [38,39,40,41].

Given the described involvement in hepatic metabolism and liver regeneration, we hypothesized that leptin serum concentrations could serve as a diagnostic biomarker for the differentiation of ALF vs. ACLF and may be related to prognosis in critically ill patients with acute liver dysfunction. This study aims to investigate the potential role of leptin as a biomarker in critically ill patients presenting with acute hepatic disease.

## 2. Materials and Methods

### 2.1. Study Design

This observational cohort study aimed to investigate the role of serum leptin in critically ill patients with acute deterioration of liver function. From 2015 until 2021, we prospectively enrolled 160 patients with advanced liver disease or acute liver failure from our medical intensive care unit (ICU) in the Department of Gastroenterology, Digestive Disease, and Intensive Care Medicine at the University Hospital RWTH Aachen. Written informed consent was obtained from the patient, their spouse, or an appointed legal guardian. Inclusion criteria were patients above the age of 18 years with known or suspected chronic liver disease or acute liver failure. Exclusion criteria were an expected short-term treatment (less than 48 h) on the ICU, acute poisoning, or pregnancy. The diagnoses of ALF or ACLF were based on the latest definitions according to the guidelines of the European Association for the Study of the Liver (EASL) [1,2]. To collect follow-up data on patient survival, we contacted the patient, their relatives, or their primary care physician, provided consent was available. As a control group to critically ill patients, we collected blood samples from 20 healthy volunteers from the local blood bank with regular values in blood counts as well as absent signs of acute infection or relevant chronic disease in clinical examination. The study was conducted in agreement with the 1964 Declaration of Helsinki and was approved by the local ethics committee (EK 150/06) of the University Hospital RWTH Aachen.

### 2.2. Leptin Measurements

We collected serum blood samples on the day of ICU admission as well as 48 h afterward. After centrifugation for 10 min and aliquotation, the serum samples were kept frozen at −80 °C until further investigation. Serum concentrations of leptin were measured using a commercially available enzyme-linked immunosorbent assay (ELISA) kit according to the manufacturer’s instructions (R&D systems Europe, 19 Barton Lane, Abingdon Science Park, Abingdon OX14 3NB, UK). Measurements were performed blinded to clinical or other laboratory data.

### 2.3. Statistical Analysis

For statistical analysis and visualization, we used SPSS version 29 (SPSS, Chicago, IL, USA) and Python version 3.11 [42]. Data are presented as median and range due to the skewed distribution of most parameters. Since normality could not be assumed, we employed the Mann–Whitney U test or chi-squared test for comparing two groups and the Kruskal–Wallis test followed by a Dunn’s multiple comparison test, or the chi-squared test for more than two groups. For paired sample comparisons, we used the Wilcoxon signed rank test. A significance level of α = 0.05 was used for all statistical calculations. To evaluate correlations between parameters, Spearman’s rank correlation test was used. Uni- and multivariable linear regression models were calculated to further investigate the influence of covariates after correlation analysis. The association with survival was assessed using the Cox proportional hazards model. To evaluate the diagnostic performance of the biomarker, a receiver operating characteristics (ROC) curve and the corresponding area under the curve (AUC) were used in conjunction with test performance measures including sensitivity, specificity, positive predictive value (PPV), and negative predictive value (NPV).

## 3. Results

### 3.1. Serum Levels of Leptin Are Elevated in Critically Ill Patients Presenting with Acute Liver Failure

We enrolled 160 critically ill patients from the intensive care unit (ICU) in the study between the years of 2015 and 2021. Of these patients, 20 presented with dACLD, 123 with ACLF, and 17 with ALF. Although we did not observe a statistical difference in median age between the patient cohorts, patients with ALF seemed to be slightly younger, with a median age of 52, compared to dACLD (median age of 59.5) and ACLF (median age of 58). Critically ill patients admitted due to ACLF showed a higher body mass index (BMI) with a median of 27.6 compared to patients with dACLD (median of 23.1) and ALF (median of 25.7). Disease severity and organ failure were much higher among patients with ACLF compared to dACLD and ALF as represented by the acute physiology and chronic health evaluation II score (APACHE II) with a median of 27 in ACLF, 16.5 in dACLD, and 15 in ALF (*p* < 0.001); sequential organ failure assessment score (SOFA) with a median of 14 in ACLF, 8 in dACLD, and 9 in ALF (*p* < 0.001); need for mechanical ventilation (52% in ACLF, 15% in dACLD, and 11.8% in ALF, *p* < 0.001); and vasopressor demand (81.3% in ACLF, 5% in dACLD, and 23.5% in ALF, *p* < 0.001). The model of end-stage liver disease (MELD) score was higher in ACLF and ALF compared to dACLD, with a median of 28 in ACLF, 30 in ALF, and 13 in dACLD (*p* < 0.001). The length of stay in the ICU was longer in patients with ACLF and ALF (median of 6 days in ACLF and ALF, vs. 3 days in dACLD, *p* < 0.001). This consequently led to higher mortality rates in the ACLF group in the ICU (56.9% in ACLF, 0% in dACLD, and 23.5% in ALF, *p* < 0.001) and overall (1-year mortality 67.3% in ACLF, 15.8% in dACLD, and 31.2% in ALF, *p* < 0.001). Liver transplantation (LT) was more frequent in the ALF group compared to dACLD and ACLF (35.5% in ALF, 15% in dACLD, and 9.8% in ACLF, *p* = 0.014) (Table 1).

**Figure 1:**
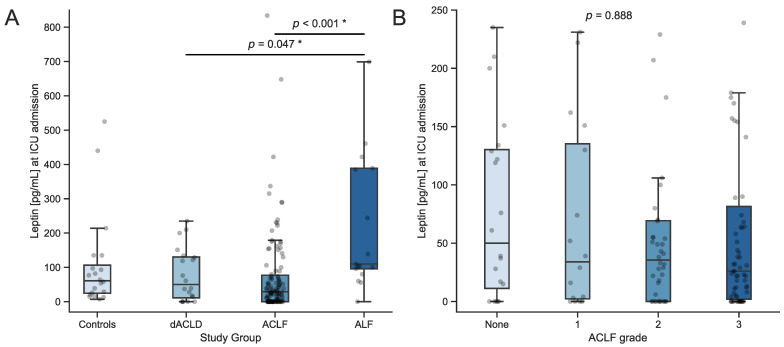
(**A**) Serum leptin concentrations in healthy controls compared to critically ill patients with dACLD, ACLF, and ALF. (**B**) Comparison between ACLF grades in ACLF patients. Sample sizes: controls *n* = 20, dACLD *n* = 20, ACLF *n* = 123, ALF *n* = 17. Overlaid dots represent individual measurements. * Significance between groups was assessed using the Kruskal–Wallis test followed by Dunn’s multiple comparison test. *p*-values < 0.05 were considered statistically significant and were highlighted (“*”). Abbreviations: dACLD: decompensated advanced chronic liver disease; ACLF: acute-on-chronic liver failure; ALF: acute liver failure.

Median serum leptin concentrations were similar in healthy controls, dACLD, and ACLF. However, median serum leptin levels were significantly higher in ALF compared to dACLD (110 vs. 50 pg/mL, *p* = 0.047) or ACLF (110 vs. 29 pg/mL, *p* < 0.001) (Figure 1A). Serum leptin tends to be lower in higher grade ACLF, although this difference did not reach statistical significance (Figure 1B).

**Figure A1:**
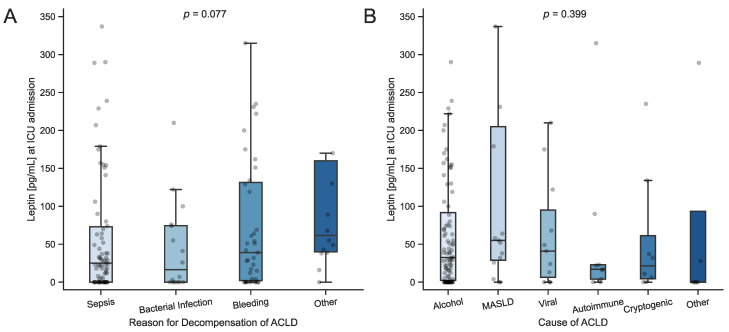
(**A**) Serum leptin concentrations at ICU admission compared between different reasons for decompensation of ACLD in dACLD and ACLF patients. (**B**) Comparison of serum leptin levels between different causes of ACLF in dACLD and ACLF patients. Sample sizes: dACLD *n* = 21, ACLF *n* = 124. Overlaid dots represent individual measurements. Significance between groups was assessed using the Kruskal–Wallis test. *p*-values < 0.05 were considered statistically significant. Abbreviations: ACLD: (decompensated) advanced chronic liver disease; ACLF: acute-on-chronic liver failure; MASLD: metabolic dysfunction-associated steatotic liver disease.

Among critically ill patients admitted to the ICU due to dACLD or ACLF, 74 (51.7%) were admitted due to sepsis without acute liver dysfunction, 39 (27.3%) due to gastrointestinal or other bleeding, 16 (11.2%) due to nonseptic bacterial infection, and 14 (9.8%) for other reasons. In the next step, we compared leptin serum concentrations at ICU admission between these groups. While patients with dACLD or ACLF admitted due to sepsis or bacterial infections tended towards lower serum leptin concentrations compared to those admitted for bleeding or other reasons, this difference did not reach statistical significance (Figure A1A). Additionally, we compared leptin serum concentrations at ICU admission between different causes of chronic liver disease in dACLD or ACLF patients admitted to the ICU. Patients with metabolic dysfunction-associated steatotic liver disease (MASLD) showed a trend towards higher levels of serum leptin compared to other patients, but this trend also did not reach statistical significance (Figure A1B).

### 3.2. Low Serum Leptin at ICU Admission Reliably Rules out Acute Liver Failure in Critically Ill Patients with Liver Dysfunction

**Figure 2:**
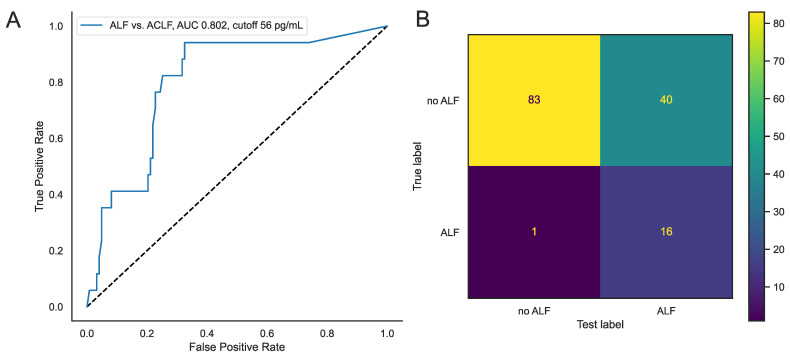
(**A**) Receiver operating characteristic (ROC) curves for ALF diagnosis using serum leptin levels at ICU admission in patients with ACLF. (**B**) Confusion matrix for the diagnosis of ALF using serum leptin levels in patients with acute or acute-on-chronic liver failure. The cutoff value was determined by the Youden index for critically ill patients with acute (-on-chronic) liver failure. The sample size included 140 patients. Abbreviations: AUC: area under curve; ACLF: acute-on-chronic liver failure; ALF: acute liver failure.

Given the elevation of serum leptin in critically ill patients with ALF admitted to the ICU, we evaluated the diagnostic capabilities of serum leptin at ICU admission in differentiating between ALF and ACLF. In a receiver operating characteristic (ROC) curve analysis, serum leptin at ICU admission showed an area under the curve (AUC) of 0.802 (95% CI 0.695–0.909), with a cutoff at 56 pg/mL for diagnosing of ALF (Figure 2A). A confusion matrix using this cutoff revealed only one patient incorrectly diagnosed as not having ALF who presented with ALF and 16 correctly diagnosed ALF patients. Among patients without ALF, 83 were correctly diagnosed, whereas 40 patients who were diagnosed with ALF were admitted due to ACLF (Figure 2B). This analysis results in a sensitivity of 94.1% and a specificity of 67.5%. The positive predictive value lies at 28.6%, while the negative predictive value was calculated at 98.8% (Table 2).

### 3.3. Serum Levels of Leptin at ICU Admission Correlate with Body Mass Index, Hemoglobin, Inflammation, and Aminotransferases in Critically Ill Patients with Liver Dysfunction

To detect and evaluate other possible influences and covariates among clinical and laboratory parameters, we conducted extensive correlation analyses of those parameters with serum leptin. In this analysis, we found a moderately strong positive correlation between serum leptin and BMI (Spearman’s r 0.280, *p* < 0.001). Male patients exhibited higher levels of serum leptin concentrations compared to female patients (median of 92 vs. 68 pg/mL, *p* = 0.019). Among markers of inflammation, C-reactive protein (CRP) and interleukin 6 (IL-6) showed moderately strong negative correlations with serum leptin (Spearman’s r −0.303, *p* < 0.001, and Spearman’s r −0.229, *p* = 0.011, respectively). However, procalcitonin (PCT) did not exhibit any correlation with serum leptin levels (Spearman’s r −0.109, *p* = 0.191). Hemoglobin (Hb) was positively correlated with serum leptin (Spearman’s r 0.271, *p* < 0.001). Additionally, we observed a moderately strong positive correlation between aminotransferases and serum leptin (AST: Spearman’s r 0.210, *p* = 0.008; ALT: Spearman’s r 0.258, *p* = 0.001). Lastly, the N-terminal prohormone of brain natriuretic peptide (NT-proBNP) was negatively correlated with serum leptin (Spearman’s r −0.315, *p* < 0.001), while lactate was positively correlated (Spearman’s r 0.289, *p* < 0.001). Importantly, we did not find any correlations with disease severity or measures of mortality (Table 3).

**Figure 3:**
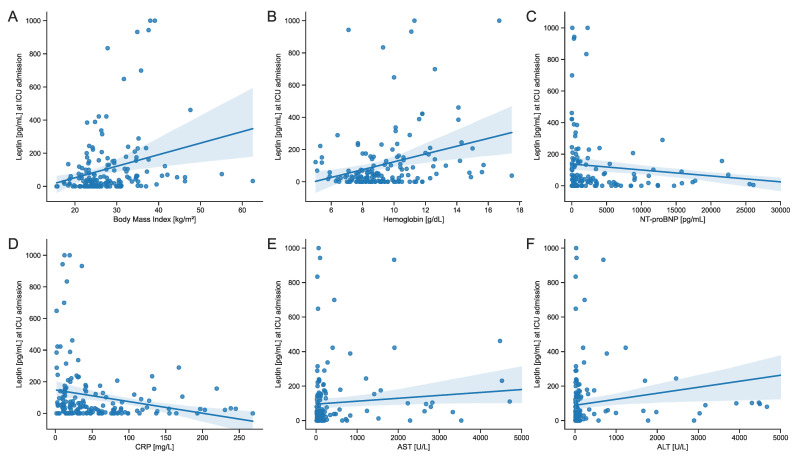
Scatter plots with a plotted linear regression fit between serum leptin concentrations at ICU admission and clinical parameters in all patients: (**A**) Body mass index, (**B**) hemoglobin, (**C**) NT-proBNP, (**D**) CRP, (**E**) AST, and (**F**) ALT. Regression analyses were conducted using univariate linear regression (see Table 4 for coefficients and *p*-values). The shaded areas in the plots represent the 95% confidence interval for the regression estimate. Abbreviations: NT-proBNP: N-terminal prohormone of brain natriuretic peptide; CRP: C-reactive protein; AST: aspartate aminotransferase; ALT: alanine aminotransferase.

To further examine the relationship between and within the correlated parameters to serum leptin, we conducted uni- and multivariable regression analyses. In these analyses, BMI, ALT, and hemoglobin showed the strongest positive influence, with beta coefficients of 50.22, 57.72, and 56.17, respectively. Conversely, CRP and NT-proBNP were the strongest negative predictors, with beta coefficients of −41.09 and −38.91. In the multivariable regression analysis, only BMI and Hb remained as relevant predictors for serum leptin concentrations (Figure 3 and Table 4).

Several chronic diseases and conditions were analyzed for their influence on serum leptin levels through uni- and multivariable regression analyses. Patients without diabetes mellitus or chronic alcohol consumption displayed higher serum concentrations of leptin. This finding was consistent in the multivariable analysis (Table 5).

### 3.4. Levels of Serum Leptin Are Stable in the Early Stage of Critical Illness in Patients with Liver Disease

**Figure 4:**
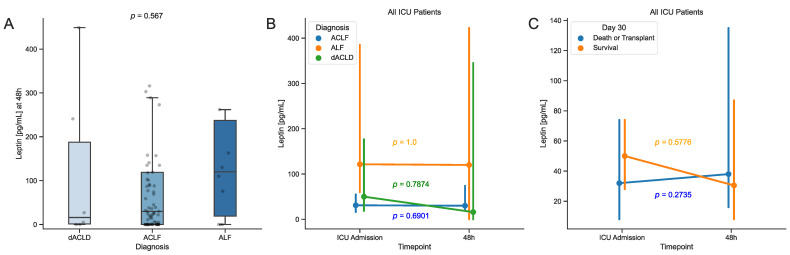
(**A**) Serum leptin levels after 48 h of treatment on the ICU in the respective patient cohorts. Overlaid dots represent individual measurements. (**B**) Comparison of serum leptin concentrations at ICU admission to levels after 48 h of treatment in between the patient cohorts. (**C**) Comparison of serum leptin concentrations at ICU admission to levels after 48 h of treatment between surviving vs. deceased or transplanted patients at day 30. Vertical error bars indicate the 95% confidence intervals around the median (**B**,**C**). Sample sizes: patients *n* = 85. Significance between groups was assessed using the Kruskal-Wallis test for more than two groups or the Wilcoxon signed rank test for paired samples. *p*-values < 0.05 were considered statistically significant. Abbreviations: ICU: intensive care unit; dACLD: decompensated advanced chronic liver disease; ACLF: acute-on-chronic liver failure; ALF: acute liver failure.

To shed light on the initial regulations of serum leptin concentrations and their implications in critically ill patients admitted to the ICU due to liver disease, we also measured follow-up concentrations of serum leptin in 85 patients who were available for sampling after 48 h of ICU treatment. When comparing the serum leptin concentrations after 48 h of ICU treatment between the patient cohorts, we observed a similar pattern to the levels at ICU admission. Patients with dACLD and ACLF showed the lowest median levels of serum leptin, while patients with ALF showed a higher median (Figure 4A). However, this difference did not reach statistical significance. In a paired analysis comparing the serum leptin levels at ICU admission to levels after 48 h of ICU treatment, we again did not observe any significant changes in the patient cohorts (Figure 4B). To elucidate a possible influence of the initial regulation of serum leptin concentrations on survival, we compared serum leptin levels upon ICU admission to levels after 48 h of treatment between surviving and deceased or transplanted patients at day 30. Although not showing statistical significance, surviving patients had a tendency towards lower serum leptin levels after 48 h of treatment, while deceased or transplanted patients did not (Figure 4C).

## 4. Discussion

In this study, we investigated the diagnostic and prognostic impact of serum leptin concentrations in critically ill patients admitted to the intensive care unit (ICU) due to liver disease. Patients with acute liver failure (ALF) had significant higher concentrations of serum leptin upon ICU admission compared to patients with decompensated advanced liver disease (dACLD) or acute-on-chronic liver failure (ACLF). Strikingly, a serum leptin level below 56 pg/mL could effectively rule out ALF in our patient cohort with a negative predictive value (NPV) of 98.8%. Analysis of potential covariates of serum leptin indicated that BMI and hemoglobin (Hb) were the most significant covariates. Moreover, our data showed no association between serum leptin concentrations and transplant-free survival in critically ill patients with liver disease. Furthermore, serum leptin levels remained consistent during the initial 48 h of ICU treatment and did not exhibit any statistically significant differences between patient cohorts or when comparing survivors, deceased, or transplanted patients at day 30 post-ICU admission.

Leptin is secreted by adipose tissue primarily to regulate energy intake by signaling satiety to the central nervous system [10,20], but it has also gained interest in liver disease and critical illness. Regulated hepatic functions include insulin sensitivity, immune response, inflammation, angiogenesis, fibrogenesis, and tissue remodeling [24,26]. Leptin has been linked to metabolic dysfunction-associated steatotic liver disease (MASLD) and its severity [29,32,33], and has been shown to play a role in the hepatic fibrogenic response to chronic liver injury and liver tissue regeneration [30,35]. Serum leptin was found to be decreased in liver cirrhosis with higher depletion in higher-grades and association to fat mass [43,44]. Although not reaching statistical significance, we also observed higher levels of serum leptin in patients with MASLD (Figure 1B). Most notably, we found elevated levels of serum leptin in critically ill patients with ALF compared to dACLD and ACLF (Figure 1A). Our data are in line with previously published data that opened the hypothesis of leptin being a relevant factor in acute hepatic tissue injury and regulator in liver regeneration. Following a toxic injury, leptin-deficient mice showed impaired liver regeneration [35]. Additionally, in humans after hepatectomy, serum leptin levels on the first postoperative day have been shown to be positively correlated to high liver regeneration [37]. Moreover, an intraperitoneal injection of leptin was found to increase liver regeneration in rats that had undergone 70% hepatectomy [36]. The elevated leptin concentrations in our patient cohort with ALF might indicate or even result in liver regeneration that is more pronounced in ALF than ACLF patients. Low serum leptin levels likely exclude ALF with a high negative predictive value (Table 2). However, high serum leptin levels also occur in about one-third of patients with ACLF (Figure 2B). This causes a rather low positive predictive value, making serum leptin not suitable for confirming the diagnosis of ALF (Table 2). Next to our presentation of leptin as a relevant biomarker in the differentiation between ALF and ACLF, leptin could also be a potential therapeutic target to stimulate liver regeneration in cases of acute hepatic injury, as demonstrated in a rat model [36].

**Figure A2:**
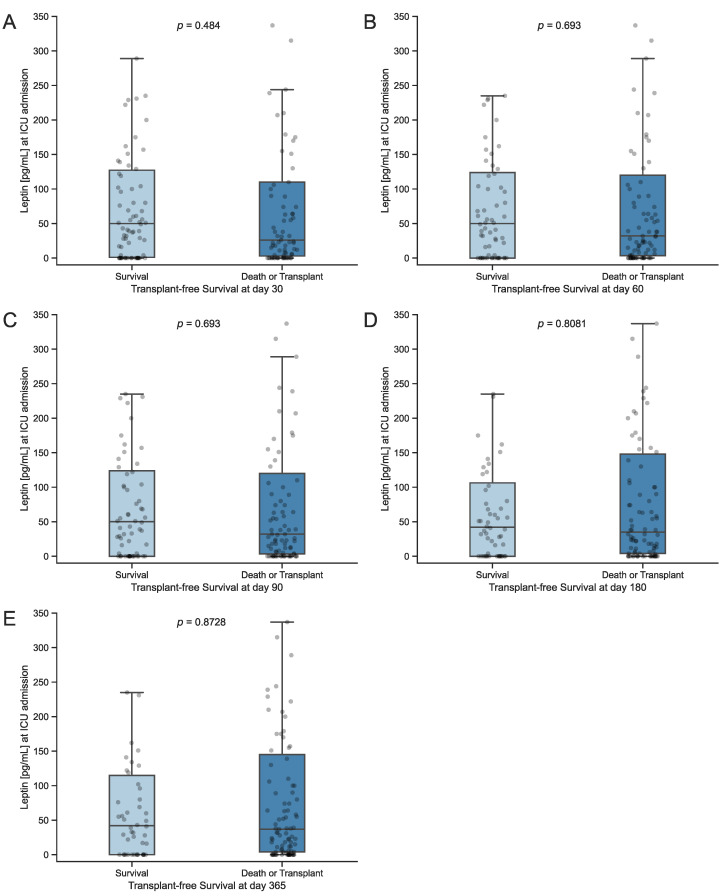
(**A**–**E**) Serum leptin levels at admission to the ICU in a consecutive survival analysis of critically ill patients treated on the ICU for survival status on days 30 through 365. Sample sizes: patients *n* = 160. Overlaid dots represent individual measurements. Significance between groups was assessed using the Mann–Whitney U test. *p*-values < 0.05 were considered statistically significant.

In critical illness in general, serum leptin could be of interest due to its involvement in the inflammatory process and glucose homeostasis [38,39,40]. As common features of critically ill patients, hyperglycemia and insulin resistance have been identified as adverse prognosis predictors in critical illness [45,46]. In fact, increased plasma leptin concentrations have been found after intracerebral hemorrhage and was associated with a poor clinical outcome [47]. However, in other critical illness such as sepsis, the picture is less clear with conflicting evidence [41,48]. Regarding the influence of nutritional status and sex, we also observed a positive correlation between BMI and serum leptin, with higher serum levels in female patients, as previously described [13]. Somewhat unexpectedly, we found a correlation with hemoglobin (Hb), which has not been described before and contradicts the notion that male patients have higher hemoglobin levels (given that leptin is lower in males). Our regression analysis showed lower levels of serum leptin in individuals with diabetes mellitus, which may be biased by the high levels of serum leptin in patients with ALF without diabetes, as the opposite has been described [49]. In our study, we did not observe any differences in serum leptin when comparing surviving, deceased, or transplanted patients (Figure A2). However, our cohort of patients with ALF was too small to exclude an influence on survival in this patient cohort. Interestingly, our results indicate that serum leptin is relatively stable in the first 48 h of ICU treatment, supporting its use as a biomarker (Figure 4).

It is important to note the most relevant limitations of this study. A single-center study provides high technical accuracy and reproducibility but lacks the opportunity to include a wider variety of patients. Particularly in the smaller patient subgroups (dACLD and ALF), analyses are somewhat limited and may lack statistical power. Since some measurements were at the upper cutoff of 1000 pg/mL, dilution could potentially provide a better understanding and improve statistical analysis. Extensive correlation analyses must be carefully interpreted within the clinical context to avoid false positives. Future studies are needed to confirm our results in other patient cohorts.

## 5. Conclusions

This study reveals that critically ill patients admitted to the intensive care unit (ICU) due to acute liver failure (ALF) have elevated levels of serum leptin compared to patients with decompensated advanced liver disease (dACLD) or acute-on-chronic liver failure (ACLF). This may indicate acute tissue injury with ongoing hepatic remodeling. Interestingly, low serum levels of leptin could be used to rule out ALF in patients with acute hepatic disease with a high negative predictive value (NPV), but not to confirm it. Additionally, our study found no association between serum leptin levels and transplant-free survival. Serum leptin levels remained stable within the first 48 h of ICU treatment, regardless of the category of hepatic disease or transplant-free survival.

## Figures and Tables

**Table 1:** Baseline patient characteristics.

Parameter	dACLD	ACLF	ALF	*p*
Number, *n*	20	123	17	
Sex (female/male), *n*	9/11	50/73	9/8	0.612
Age, [years]	59.5 (24–80)	58 (19–87)	52 (24–78)	0.196
BMI, [kg/m^2^]	23.1 (15.8–41.5)	27.6 (15.6–62.5)	25.7 (21.7–47.6)	0.005 *
APACHE II score	16.5 (5–37)	27 (8–55)	15 (7–39)	<0.001 *
SOFA score	8 (3–15)	14 (5–20)	9 (4–18)	<0.001 *
MELD score	13 (7–28)	28 (8–40)	30 (20–40)	<0.001 *
Liver transplantation, *n* (%)	3 (15.0)	12 (9.8)	6 (35.5)	0.014 *
Mechanical ventilation, *n* (%)	3 (15.0)	64 (52.0)	2 (11.8)	<0.001 *
Vasopressor demand, *n* (%)	1 (5.0)	100 (81.3)	4 (23.5)	<0.001 *
ICU days *n*	3 (2–15)	6 (1–178)	6 (3–39)	<0.001 *
Death on ICU, *n* (%)	0 (0)	70 (56.9)	4 (23.5)	<0.001 *
30-day mortality, *n* (%)	0 (0)	60 (52.6)	2 (11.8)	<0.001 *
1-year mortality, *n* (%)	3 (15.8)	76 (67.3)	5 (31.2)	<0.001 *
Leptin at ICU admission, [pg/mL]	50 (0–235)	29 (0–1000)	110 (0–1000)	<0.001 *

The median and range (in parentheses) are given unless otherwise indicated. Abbreviations: dACLD: decompensated advanced chronic liver disease; ACLF: acute-on-chronic liver failure; ALF: acute liver failure; APACHE II: acute physiology and chronic health evaluation; SOFA: sequential organ failure assessment; MELD: model of end stage liver disease; ICU: intensive care unit. Significance between dACLD, ACLF and ALF patients was assessed using the Kruskal-Wallis test or chi-squared test, respectively. *p*-values < 0.05 were considered statistically significant and highlighted (“*”).

**Table 2:** Performance metrics for the diagnosis of ALF by serum leptin concentration at ICU admission.

Metric	Value (%)
Sensitivity	94.1
Specificity	67.5
PPV	28.6
NPV	98.8

Abbreviations: PPV: positive predictive value; NPV: negative predictive value.

**Table 3:** Correlations of clinical and laboratory parameters with leptin serum concentrations at ICU admission.

Parameter	Spearman’s r	*p*
Demographics		
Age	0.099	0.213
Body mass index	0.289	<0.001 *
Blood count and markers of inflammation		
Leukocytes	0.068	0.396
Hemoglobin	0.271	0.001 *
Platelets	0.067	0.401
C-reactive protein	−0.303	<0.001 *
Procalcitonin	−0.109	0.191
Interleukin 6	−0.229	0.011 *
Electrolytes and renal system		
Sodium	0.037	0.646
Potassium	−0.006	0.943
pH	0.083	0.296
Urea	−0.176	0.026 *
Creatinine	−0.134	0.091
eGFR	0.117	0.142
Diuresis per day	0.092	0.258
Hepato-pancreatico-biliary system and coagulation		
Albumin	0.143	0.072
INR	0.098	0.22
Bilirubin, total	0.050	0.529
AST	0.210	0.008 *
ALT	0.258	0.001 *
γGT	0.046	0.567
AP	0.021	0.790
AFP	0.125	0.553
Cholesterol, total	0.095	0.245
Triglycerides	0.048	0.552
Cardiopulmonary system		
NT-proBNP	−0.315	<0.001 *
Norepinephrine demand	−0.124	0.207
Horovitz quotient (PaO_2_/FiO_2_)	0.142	0.073
FiO_2_	−0.127	0.11
Lactate	0.289	<0.001 *
Disease severity and clinical scores		
Length of stay in ICU	0.077	0.331
Length of stay in hospital	−0.061	0.442
SOFA score	−0.078	0.329
APACHE II score	−0.031	0.701
SAPS II score	−0.102	0.197
MELD	0.047	0.553
Child-Pugh points	−0.025	0.765
CLIF-C OF score	−0.116	0.178
CLIF-C ACLF score	0.060	0.508

* Spearman’s rank correlation test was used to calculate correlations of positive and negative nature. *p*-values < 0.05 were considered statistically significant and were highlighted (“*”). Abbreviations: eGFR: estimated glomerular filtration rate; INR: international normalized ratio; AST: aspartate aminotransferase; ALT: alanine aminotransferase; γGT: gamma-glutamyltransferase; AP: alkaline phosphatase; AFP: alpha-fetoprotein; NT-proBNP: N-terminal prohormone of brain natriuretic peptide; FiO_2_: fraction of inspired oxygen; ICU: intensive care unit; SOFA: sequential organ failure assessment; APACHE-II: acute physiology and chronic health evaluation II; SAPS II: simplified acute physiology score II; MELD: model of end stage liver disease; CLIF-C OF: chronic liver failure consortium (CLIF-C) organ failure score; CLIF-C ACLF: chronic liver failure consortium (CLIF-C) ACLF score; ACLF: acute-on-chronic liver failure.

**Table 4:** Uni- and multivariable linear regression analyses of serum leptin concentrations at ICU admission in all patients to demographics and laboratory parameters.

	Univariable Regression				Multivariable Regression		
Covariate	Beta Coefficient	Coefficient	95% CI	*p*	Coefficient	95% CI	*p*
BMI	50.22	6.973	2.960–10.986	<0.001 *	7.278	3.018–11.539	<0.001 *
Male sex	−24.67	−49.914	−109.806–9.978	0.102			
Hemoglobin	56.17	24.256	11.937–36.576	<0.001 *	24.831	9.164–40.498	0.002 *
CRP	−41.09	−0.738	−1.261–−0.214	0.006 *	−0.590	−1.180–−0.001	0.050
IL-6	−5.23	0.000	−0.002–0.001	0.778			
Urea	−13.89	−0.215	−0.675–0.246	0.358			
AST	35.92	0.017	0.003–0.031	0.017 *	−0.004	−0.033–0.025	0.786
ALT	57.72	0.035	0.018–0.052	<0.001 *	0.019	−0.016–0.055	0.284
NT-proBNP	−38.91	−0.004	−0.007–−0.001	0.026 *	−0.002	−0.005–0.002	0.360
Lactate	21.80	6.713	−2.42–15.85	0.149			

Beta coefficients represent standardized coefficients for comparability. * Significance was assessed using a linear regression model. *p*-values < 0.05 were considered statistically significant and were highlighted (“*”). Abbreviations: BMI: body mass index; CRP: C-reactive protein; IL-6: interleukin 6; AST: aspartate aminotransferase; ALT: alanine aminotransferase; NT-proBNP: N-terminal prohormone of brain natriuretic peptide.

**Table 5:** Uni- and multivariable linear regression analyses for comorbidities as covariates of serum leptin at ICU admission.

	Univariable Regression			Multivariable Regression		
Covariate	Coefficient	95% CI	*p*	Coefficient	95% CI	*p*
Diabetes mellitus	71.1	4.1–138.0	0.038 *	77.5	11.6–143.4	0.021 *
Hypertension	−38.6	−103.9–26.7	0.245			
Coronary artery disease	−16.3	−108.6–75.9	0.727			
Chronic alcohol consumption	72.1	13.3–130.9	0.017 *	77.1	19.0–135.3	0.010 *
Chronic obstructive pulmonary disease	81.1	−31.6–193.7	0.157			
Malignancy	79.9	−7.6–167.5	0.073			
Hepatocellular carcinoma	54.8	−81.9–191.6	0.43			
Hematological neoplasm	63.5	−93.4–220.3	0.425			
Solid neoplasm	75.5	−70.0–221.0	0.307			

Coefficients are calculated for the absence of the respective disease or risk factor. * Significance was assessed using a linear regression model. *p*-values < 0.05 were considered statistically significant and were highlighted (“*”).

## Data Availability

The original data sets presented in this study are available on reasonable request from the corresponding author.

## Appendix A

### Appendix A.1. Serum Leptin Is Not Predictive for Survival in Critically Ill Patients with Liver Dysfunction

Additionally, we focused on elucidating the prognostic value of serum leptin concentrations at ICU admission in critically ill patients with liver disease. Initially, we conducted a univariate Cox regression analysis for serum leptin in relation to time to death or transplant. Here, we did not observe any influence of serum leptin levels on transplant-free survival (HR 0.999, 95% CI 0.999–1.001, *p* = 0.854). Similarly, when comparing serum levels of leptin at ICU admission between surviving and deceased or transplanted patients at various time points (i.e., after 30, 60, 90, 180, and 365 days), we also did not observe statistically significant differences. Comparing serum leptin levels at ICU admission in survivors to non-survivors of the ICU also yielded no difference (*p* = 0.314). However, median serum leptin levels were consistently slightly lower in deceased or transplanted patients (Figure A2). In the subgroup analyses of ALF and ACLF patients, serum leptin levels were also not associated with survival.
